# New Insights into the Pathophysiology of Coronary Artery Aneurysms

**DOI:** 10.3390/diagnostics14192167

**Published:** 2024-09-29

**Authors:** Iris Bararu-Bojan, Oana-Viola Badulescu, Minerva Codruta Badescu, Maria Cristina Vladeanu, Carmen Elena Plesoianu, Andrei Bojan, Dan Iliescu-Halitchi, Razvan Tudor, Bogdan Huzum, Otilia Elena Frasinariua, Manuela Ciocoiu

**Affiliations:** 1 Department of Pathophysiology, University of Medicine and Pharmacy Grigore T. Popa, 700115 Iasi, Romania; iris.bararu@umfiasi.ro (I.B.-B.); manuela.ciocoiu@umfiasi.ro (M.C.); 2 Department of Internal Medicine, University of Medicine and Pharmacy Grigore T. Popa, 700115 Iasi, Romania; minerva.badescu@umfiasi.ro (M.C.B.); carmen-elena.plesoianu@umfiasi.ro (C.E.P.);; 3 Department of Surgical Sciences, University of Medicine and Pharmacy Grigore T. Popa, 700115 Iasi, Romania; 4 Department of Pediatry, University of Medicine and Pharmacy Grigore T. Popa, 700115 Iasi, Romania

**Keywords:** coronary artery aneurysms, aneurysms, vascular remodelling

## Abstract

Coronary aneurysms are typically defined as sections of a coronary artery where the diameter is more than 1.5 times that of an adjacent normal segment. In rare circumstances, these aneurysms can become exceedingly large, leading to the classification of giant coronary artery aneurysms. Despite their occurrence, there is no clear consensus on the precise definition of giant coronary artery aneurysms, and their etiology remains somewhat ambiguous. Numerous potential causes have been suggested, with atherosclerosis being the most prevalent in adults, accounting for up to 50% of cases. In pediatric populations, Kawasaki disease and Takayasu arteritis are the primary causes. Although often discovered incidentally, coronary artery aneurysms can lead to severe complications. These complications include local thrombosis, distal embolization, rupture, and vasospasm, which can result in ischemia, heart failure, and arrhythmias. The optimal approach to medical, interventional, or surgical management of these aneurysms is still under debate and requires further clarification. This literature review aims to consolidate current knowledge regarding coronary artery aneurysms’ pathophysiology, emphasizing their definition, causes, complications, and treatment strategies. Recent research has begun to explore the molecular mechanisms involved in the formation and progression of coronary artery aneurysms. Various molecules, such as matrix metalloproteinases (MMPs), inflammatory cytokines, and growth factors, play crucial roles in the degradation of the extracellular matrix and the remodeling of vascular walls. Elevated levels of MMPs, particularly MMP-9, have been associated with the weakening of the arterial wall, contributing to aneurysm development. Inflammatory cytokines such as tumor necrosis factor-alpha (TNF-α) and interleukins (IL-1β and IL-6) have been implicated in promoting inflammatory responses that further degrade vascular integrity. Additionally, growth factors such as vascular endothelial growth factor (VEGF) may influence angiogenesis and vascular remodeling processes. Understanding these molecular pathways is essential for developing targeted therapies aimed at preventing the progression of coronary artery aneurysms and improving patient outcomes.

## 1. Introduction

Coronary artery aneurysms refer to localized expansions in the coronary artery that are at least 1.5 times greater in diameter than the adjacent normal segments. These aneurysms can pose significant health risks by disrupting normal blood flow and potentially leading to severe cardiac events.

On the other hand, coronary artery ectasias manifest as widespread, diffuse dilatations of the arterial segments, where the dilated length is more than 50% greater than the normal artery diameter. This distinction is crucial for accurate diagnosis and appropriate treatment planning [1].

Aneurysms can be further classified based on the integrity of the vessel wall into true aneurysms and false aneurysms, also known as pseudoaneurysms. Pseudoaneurysms are characterized by dilatations that involve only one or two layers of the vessel wall, unlike the typical three-layer structure. This condition arises due to a disruption of the media and external elastic membrane. Pseudoaneurysms are often the result of blunt chest trauma or invasive coronary procedures.

In contrast, true arterial aneurysms involve a fusiform or saccular dilatation that includes all three layers of the arterial wall: the intima, media, and adventitia. While true aneurysms are generally more stable, they still require close monitoring and management to prevent complications.

Moreover, some medical experts classify particularly large aneurysms, those exceeding 4 cm in diameter, as giant coronary aneurysms. These giant aneurysms are especially concerning due to their heightened risk of rupture and other severe complications [2].

Understanding the differences between these conditions and their classifications is essential for clinicians to develop effective treatment strategies and manage patient outcomes.

The incidence of coronary artery aneurysms (CAAs) generally ranges from 0.3% to 5.3%, with pooled data showing an average of 1.65%. However, giant CAAs are much rarer, with an incidence of only 0.02%. CAAs associated with congenital artery fistulae have a higher incidence rate of 5.9%. Despite these statistics, the actual prevalence of CAAs and coronary ectasia may be underestimated. Advancements in non-invasive imaging techniques such as computed tomography (CT) and magnetic resonance (MR) coronary angiography could enhance the detection of these conditions [3].

The reported incidence of CAAs varies significantly, indicating that these numbers might not fully represent the broader population. Several factors contribute to this variability, including differing angiographic criteria used to define CAAs and the varied definitions adopted by different institutions. For example, some studies calculate CAA incidence by including both aneurysmal coronary disease and ectasia, while others do not. Additionally, the diagnosis of CAAs via angiography can be subjective, with operator dependency and interobserver variability affecting the reported incidence rates [4,5].

Geographical differences in CAA studies underscore the significant genetic and environmental influences on their incidence, which will be explored further later. Generally, CAAs are less common in Asia compared to North America and Europe. For instance, in a study of 302 patients with Kawasaki disease (KD), the incidence of CAAs varied by ethnicity: 10.3% in Asian patients, 6.9% in Caucasian patients, and 1.2% in African patients. Additionally, a study conducted in India reported a notably high incidence of CAAs, ranging from 10% to 12%, highlighting the potential role of genetic and environmental factors in susceptibility [6].

The right coronary artery (RCA) is most affected by coronary artery aneurysms (CAAs), with studies reporting involvement rates between 40% and 70%. After the RCA, the left anterior descending artery (32.3%) and the left circumflex artery (23.4%) are also frequently involved, though prevalence varies depending on the study. In contrast, involvement of three coronary vessels or the left main coronary artery is much less common, occurring in approximately 3.5% of cases [7].

The nature of the aneurysms varies depending on their cause. Atherosclerotic or inflammatory CAAs tend to be multiple and affect more than one coronary artery. This is likely due to the diffuse nature of the underlying pathological processes that contribute to these types of aneurysms. In contrast, CAAs that are congenital, traumatic, or caused by dissection typically involve a single artery. These types of aneurysms often result from localized structural weaknesses or injuries rather than systemic conditions.

Atherosclerotic CAAs develop due to the accumulation of plaque within the artery walls, leading to inflammation and subsequent weakening of the vessel wall. This can result in multiple aneurysms within different segments of the coronary arteries. Similarly, inflammatory CAAs, often associated with conditions such as Kawasaki disease or other systemic vasculitis, also tend to affect multiple arteries due to widespread inflammation.

On the other hand, congenital CAAs arise from developmental anomalies in the coronary arteries, leading to localized structural defects that typically affect a single artery. Traumatic CAAs result from direct injury to the coronary artery, such as from blunt chest trauma or iatrogenic causes during medical procedures. Dissecting aneurysms occur when there is a tear in the artery wall, creating a false lumen and weakening the vessel, usually affecting a single artery due to the localized nature of the tear.

Understanding the patterns of artery involvement and the underlying causes of CAAs is crucial for accurate diagnosis, management, and treatment of these aneurysms. Advances in imaging technologies, such as CT and MR coronary angiography, continue to enhance our ability to detect and characterize these aneurysms, leading to better patient outcomes [8].

### 1.1. Diagnostic

**Figure 1:**
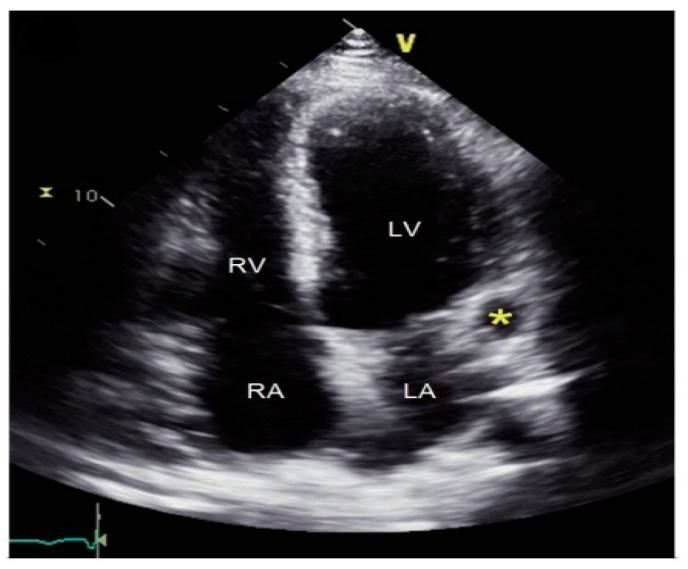
Coronary artery aneurysm—TTE image.

**Figure 2:**
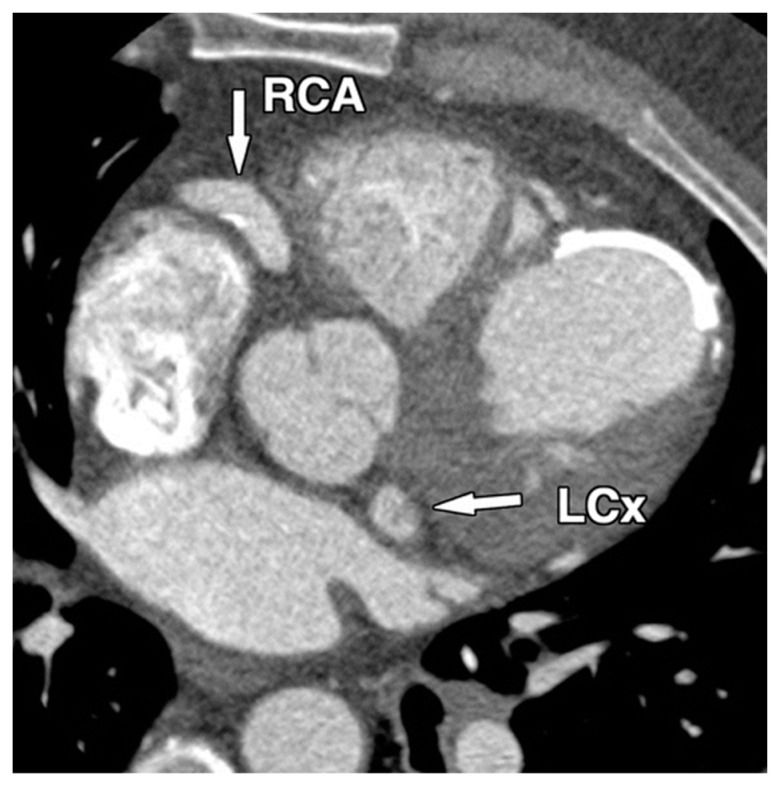
Coronary artery aneurysm—CCTA image.

Diagnosing CAAs can be challenging, as many patients remain asymptomatic or present with non-specific symptoms such as chest pain, dyspnea, or fatigue, often mimicking other cardiac conditions such as coronary artery disease. Diagnosis usually begins with non-invasive imaging techniques, such as transthoracic echocardiography (TTE), as shown in Figure 1, which can help identify the aneurysm’s size and location. More advanced imaging, including coronary computed tomography angiography (CCTA), shown in Figure 2, and magnetic resonance imaging (MRI), can provide detailed information about the extent of the aneurysm, the presence of thrombus, and any associated atherosclerosis or calcifications.

**Figure 3:**
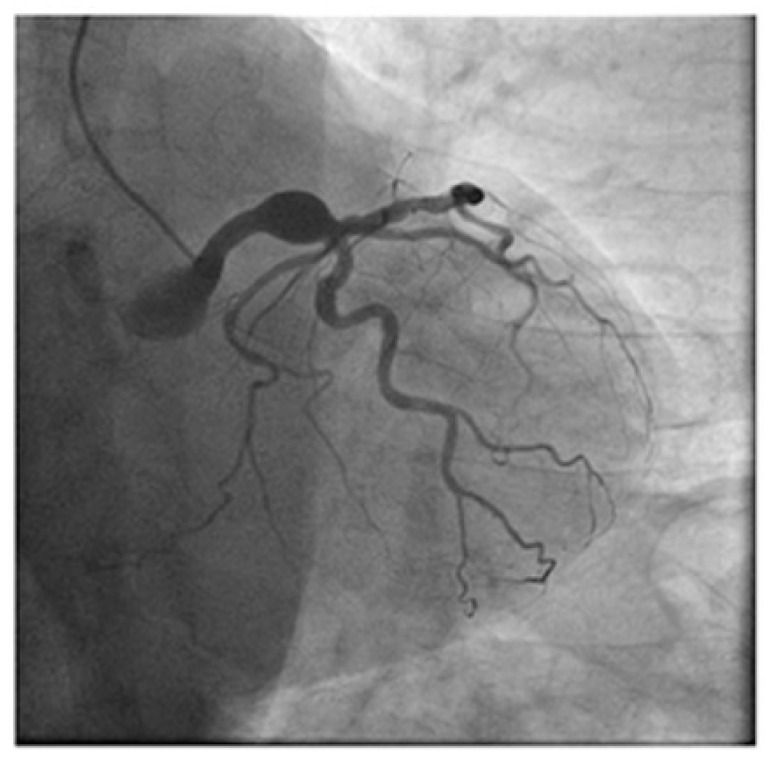
CAA aspect in coronary angiography.

In certain cases, coronary angiography is employed, especially in patients undergoing diagnostic workups for chest pain or acute coronary syndromes, where CAAs may be incidentally discovered—an example is given in Figure 3. This technique allows real-time visualization of blood flow and can help confirm the diagnosis, assess the risk of rupture, and guide treatment strategies. Additionally, cardiac biomarkers, though not specific to CAAs, may aid in diagnosing concurrent ischemia or myocardial infarction. Once diagnosed, ongoing monitoring is crucial, as CAAs are associated with risks such as thrombosis, rupture, and progression to more severe coronary artery disease.

### 1.2. Differences between Coronary Artery Aneurysms (CAAs) and Giant Coronary Artery Aneurysms (GCAAs)

Coronary artery aneurysms (CAAs) and giant coronary artery aneurysms (GCAAs) share similarities in their fundamental pathology but differ significantly in frequency and size definitions. CAAs are relatively rare, occurring in approximately 0.3–5% of patients undergoing coronary angiography, while GCAAs, defined as aneurysms exceeding 20 mm in diameter, are even more uncommon, with a frequency of less than 0.02%. Despite their rarity, both conditions are often detected incidentally during imaging studies for coronary artery disease (CAD) or other cardiac conditions. In both cases, the risk of complications, such as thrombosis, rupture, or ischemia, increases with the size of the aneurysm, making early detection critical.

In terms of etiology, CAAs and GCAAs share overlapping causes, but there are some distinctions in their underlying mechanisms. The most common cause for both conditions is atherosclerosis, which weakens the arterial wall through chronic inflammation and plaque formation. Other shared causes include Kawasaki disease (especially in pediatric cases), connective tissue disorders, vasculitis, and infections. However, GCAAs are more likely to be associated with severe or advanced forms of these conditions, where chronic or aggressive inflammatory processes lead to significant arterial damage and extreme dilation. Additionally, trauma or previous coronary interventions, such as stent placements, are more commonly linked to the development of GCAAs due to mechanical disruption of the vessel wall.

Both CAAs and GCAAs have similar clinical implications in terms of symptoms and management approaches. Patients with either condition may be asymptomatic or present with chest pain, dyspnea, or symptoms of myocardial ischemia due to compromised coronary blood flow. However, GCAAs pose a higher risk of rupture, thrombosis, or embolization due to their larger size and the increased likelihood of turbulent blood flow within the aneurysm. Consequently, while medical management with antiplatelet or anticoagulant therapy is common for both conditions, GCAAs are more frequently considered for surgical or interventional treatment, such as aneurysm resection, stenting, or bypass grafting, to prevent catastrophic complications [8,9].

### 1.3. Objectives

First, our review aims to consolidate and clarify the underlying mechanisms driving the formation and progression of CAAs, which remain poorly understood compared to other coronary artery conditions. A comprehensive review can examine factors such as inflammation, atherosclerosis, connective tissue disorders, and congenital defects, highlighting how these contribute to vessel wall weakening and aneurysmal dilation. By addressing gaps in knowledge, our review could provide insights that guide future research into targeted treatments, prevention, and risk stratification.

Secondly, the review aims to standardize the diagnostic approach to CAAs, which varies widely due to the rarity and often incidental nature of the condition. By summarizing the strengths and limitations of current diagnostic modalities—such as echocardiography, coronary computed tomography angiography (CCTA), magnetic resonance imaging (MRI), and coronary angiography—the publication would help clinicians make informed decisions about the most appropriate tools for different clinical scenarios. Additionally, it would address the importance of early detection, risk assessment, and long-term monitoring strategies, providing a clearer framework for managing CAAs to prevent complications such as thrombosis or rupture. This objective is especially crucial given the potentially severe outcomes of undiagnosed or mismanaged CAAs.

## 2. Literature Search

In our extensive narrative review, we delve deeply into the pathogenesis of coronary artery aneurysm development and detail the most significant mechanisms linked to this pathology. To achieve this, we performed a systematic search on PubMed to gather relevant studies concerning the pathogenesis of CAAs. We used specific keywords such as “coronary artery aneurysms”, “aneurysm”, and “coronary artery ectasia”.

Our search strategy was designed to include a broad range of studies to ensure comprehensive coverage of the topic. We set the following inclusion criteria: studies must have been published in English to ensure accurate interpretation and analysis; studies must have involved human subjects to ensure clinical relevance. Single case reports were excluded to focus on studies with broader applicability and robust data.

By synthesizing findings from multiple studies, our review provides valuable insights into the molecular mechanisms underlying CAAs’ pathogenesis and offers a foundation for future research aimed at developing targeted therapies and improving patient outcomes.

## 3. Pathophysiology

Atherosclerosis is the leading cause of coronary artery aneurysms (CAAs), though the precise mechanisms are not fully understood. It is thought that atherosclerosis reduces the artery’s ability to withstand intraluminal pressure, resulting in dilation. However, since only a small subset of patients with atherosclerosis develop CAAs, there may be a genetic predisposition involved. Matrix metalloproteinases (MMPs), which break down connective tissue proteins, could play a role, with the MMP3-5A allele being notably more common in patients with CAAs (*p* = 0.009).

Beyond atherosclerosis, several other conditions are associated with giant coronary artery aneurysms (GCAAs). These include congenital GCAAs; Kawasaki disease; Takayasu arteritis; and connective tissue disorders such as Marfan syndrome, Ehlers–Danlos syndrome, fibromuscular dysplasia, and neurofibromatosis. Other types of vasculitis, such as lupus, polyarteritis nodosa, Behçet’s disease, rheumatoid arthritis, ankylosing spondylitis, and scleroderma, can also result in GCAAs. In addition, infections such as HIV, bacterial and mycobacterial infections, syphilis, Lyme disease, mycotic aneurysms, and septic emboli are known to contribute.

Drug use, particularly cocaine and amphetamines, as well as certain medications such as protease inhibitors can trigger the formation of CAAs. Physical trauma to the chest, tumors, and cardiac lymphoma are also potential causes. Percutaneous coronary intervention (PCI) has been recognized as a potential trigger for aneurysm formation. The incidence of CAAs following stent implantation varies between 0.2% and 2.3%. Factors that increase the risk of CAA development after drug-eluting stent implantation include chronic total occlusion, lesion lengths greater than 33 mm, involvement of the left anterior descending artery, and implantation in an artery related to a previous infarction.

In addition to drug-eluting stents, other interventional procedures have been linked to CAA formation. These include procedures using drug-coated balloons, bioresorbable vascular scaffolds, and rotablation techniques. The mechanisms by which these procedures contribute to aneurysm formation are still being studied, but they likely involve localized damage to the arterial wall and subsequent remodeling processes.

Understanding the diverse etiologies and risk factors associated with CAAs is crucial for the effective management and prevention of these aneurysms. Continued research into the genetic and molecular pathways involved, as well as advancements in diagnostic and interventional technologies, will help improve patient outcomes and reduce the incidence of these potentially life-threatening conditions [9]—Table 1.

## 4. Atherosclerosis

CAAs are strongly associated with atherosclerosis, sharing similar histological features and clinical manifestations and often occurring together. They may represent an exaggerated form of extensive vascular remodeling in response to atherosclerotic plaque formation, with extracellular enzymatic degradation playing a key role in the development of ectatic vessels. In this process, atherosclerosis extends from the intima into the media of the arterial wall. Hyalinization and lipid deposition in the intima contribute to media degradation, primarily due to the overexpression of matrix metalloproteinases (MMPs).

MMPs are enzymes actively involved in the breakdown of extracellular matrix proteins. In the context of CAE, their overexpression results in the degradation of collagen and other structural components of the arterial wall, causing pathological dilatation. This enzymatic activity contributes significantly to the formation of ectatic vessels, highlighting the critical role of MMPs in the pathogenesis of CAAs [10].

The link between MMPs and acute coronary syndrome (ACS) further underscores the importance of these enzymes in cardiovascular diseases. Overproduction of MMPs can lead to plaque instability and rupture, precipitating ACS. This connection explains the observed beneficial effects of rosuvastatin, a cholesterol-lowering medication, which suppresses MMP expression and reduces inflammation in CAE patients. By mitigating MMP activity, rosuvastatin helps stabilize the arterial wall and prevent further dilatation.

Interestingly, the incidence may be lower in individuals with diabetes. This paradoxical observation can be attributed to the downregulation of MMP expression in diabetic patients. Diabetes appears to suppress MMP activity, thereby reducing the degradation of the extracellular matrix and the subsequent development of ectatic vessels. This phenomenon suggests a complex interplay between metabolic conditions and vascular remodeling processes.

In summary, the pathogenesis of CAAs is intricately linked to atherosclerosis through the overexpression of MMPs, leading to extensive vascular remodeling and dilatation. Understanding these mechanisms not only sheds light on the development of CAAs but also highlights potential therapeutic targets, such as MMP inhibitors, for managing this condition. Further research into the regulation of MMP activity and its impact on vascular health could pave the way for innovative treatments aimed at preventing and managing CAE and related cardiovascular diseases [11].

Thus, we can say that the most common cause of coronary artery aneurysms (CAAs) is atherosclerosis, responsible for 50% of CAAs in adults. The Coronary Artery Surgery Study (CASS) registry found a 4.9% prevalence of CAAs, higher than many other studies reporting 0.37–2.53%. The CASS analyzed over 20,000 angiograms, revealing that 1000 patients with aneurysms and coronary artery disease were more often male, had a history of myocardial infarction, and had three-vessel atherosclerotic disease. Among 978 CAA patients, 957 had coronary artery stenosis. A Greek study also found that 5.3% of 3900 angiograms showed CAAs, with 173 of 203 patients also having significant ACAD. Both studies showed no significant difference in risk factors such as hyperlipidemia, hypertension, diabetes, or smoking. Histopathological examination of atherosclerotic CAAs showed hyalinization, lipid deposition, calcification, fibrosis, and cholesterol crystals, leading to weakened vessel walls prone to aneurysm formation. Chronic inflammation and mechanical stress from stenoses may further weaken the coronary artery wall [3].

There is an association between coronary artery aneurysms (CAAs) and abdominal aortic aneurysms (AAAs), particularly through the common underlying mechanism of atherosclerosis. Atherosclerosis contributes to the weakening of the arterial walls in both the coronary and aortic arteries, leading to aneurysmal dilations. In both conditions, the process begins with chronic inflammation, endothelial dysfunction, and lipid accumulation within the arterial walls, which, over time, leads to the degradation of structural proteins such as elastin and collagen. This structural weakening, coupled with increased pressure on the vessel wall, creates the environment for aneurysm formation. Thus, patients with significant atherosclerosis are at risk of developing aneurysms in multiple vascular territories, including the coronary and aortic arteries.

While the pathophysiological link of atherosclerosis exists between CAAs and AAAs, the frequency of these conditions occurring together remains relatively low, but not insignificant. Studies have shown that patients with AAAs are more likely to have coronary artery disease, but CAAs specifically are less frequently diagnosed in the same cohort. This may be due to the differences in the mechanical forces and hemodynamics between the coronary and abdominal aortic arteries. However, when aneurysms do co-exist, they often signify widespread vascular disease, indicating a higher burden of systemic atherosclerosis. The presence of one type of aneurysm should prompt clinicians to evaluate for the other, as both conditions carry significant risks for complications such as thrombosis, rupture, and ischemia [12].

From a clinical management perspective, the co-occurrence of CAAs and AAAs in the setting of atherosclerosis can complicate treatment decisions. Patients with both aneurysm types often have a higher overall cardiovascular risk, requiring a tailored approach to management. Preventive strategies, such as aggressive lipid-lowering therapy, antiplatelet agents, and blood pressure control, are crucial to slowing the progression of atherosclerosis and reducing aneurysm growth. Surgical or interventional treatment may also need to be considered for both aneurysms, depending on their size and the risk of rupture or ischemia. Recognizing the shared atherosclerotic origin of CAAs and AAAs can help guide comprehensive cardiovascular care for affected patients [21].

### 4.1. Takayasu Arteritis

Takayasu arteritis is an inflammatory disease primarily affecting the aorta and its branches. However, approximately 10–12% of patients with Takayasu arteritis also develop coronary artery abnormalities, such as aneurysms. Unlike Kawasaki disease, which typically affects children, Takayasu arteritis usually occurs in young adults under 40. The cause of Takayasu arteritis remains unknown, with treatment options including corticosteroids, immunosuppressive therapy, biologic therapy, and occasionally surgery [8,9,10]. Diagnosing Takayasu arteritis requires a comprehensive clinical examination, which includes measuring blood pressure in both arms and legs, assessing all arterial pulses, and listening for murmurs along arterial paths. Laboratory tests to measure inflammatory markers are also essential. Imaging techniques such as MSCT-CA or magnetic resonance imaging are valuable tools, as Takayasu arteritis primarily affects the aorta and its major branches. The disease leads to the destruction of elastic fibers in the tunica media, along with significant thickening of the tunica adventitia, tunica media, and tunica intima.

### 4.2. Kawasaki Disease

Kawasaki disease is a systemic vasculitis of unknown origin that mainly affects children under 5 years old. The outcome of the disease is largely influenced by the presence of coronary lesions, including coronary artery aneurysms (CAAs). Without treatment, around 25% of patients may develop CAAs, but with proper management, this rate can be reduced to less than 5%.

In Kawasaki disease (KD), the inflammation leading to CAAs involves several pathological changes in the coronary arteries. During the acute phase of KD, the arterial wall becomes infiltrated with mononuclear cells, lymphocytes, and macrophages. This infiltration causes destruction of the internal elastic lamina, necrosis of smooth muscle cells, and myointimal proliferation, resulting in the dilation or formation of aneurysms in the coronary arteries. A crucial factor in CAA development in KD patients is the inflammatory cytokine TNF-alpha. Research has demonstrated a strong association between TNF-alpha and the pathogenesis of CAAs. In a murine model of KD, rapid production of TNF-alpha by the peripheral immune system after disease onset was found to concentrate the immune response on the coronary arteries. Elevated TNF-alpha levels were linked to the inflammatory process and elastin breakdown, contributing to CAA formation. Pharmacological intervention with etanercept, which inhibits TNF-alpha’s effects, was shown to prevent these processes in the murine model [12,21,22].

Further supporting the role of inflammation, increased expression of soluble adhesion molecules, such as vascular cell adhesion molecule-1 (VCAM-1) and intercellular adhesion molecule-1 (ICAM-1), has been observed in the sera of patients with coronary artery ectasias (CAEs). These molecules are expressed on the vascular endothelium and facilitate the migration and adherence of immune cells (leukocytes) to the coronary arteries. This process underscores the role of inflammation in the formation of CAAs, as the recruitment and adhesion of immune cells are critical steps in the inflammatory response that leads to arterial wall damage and aneurysm formation.

**Figure 4:**
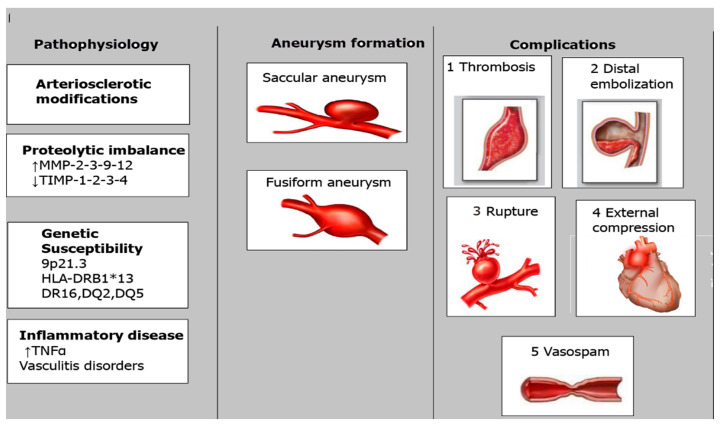
Diagram showing CAAs’ pathophysiology [23].

Understanding the inflammatory mechanisms underlying KD-associated CAAs highlights the importance of targeted anti-inflammatory therapies in managing and potentially preventing these serious cardiovascular complications [3,22]—Figure 4.

### 4.3. CAAs and Percutaneous Coronary Interventions

Recently, CAAs have been associated with percutaneous interventions such as coronary angioplasty, atherectomy, and stenting. Factors contributing to this include persistent dissection, arterial barotrauma from high-pressure balloon inflation, and the use of oversized balloons or stents, as well as atherectomy and laser angioplasty techniques. Reports show that aneurysm rates are 3.9% following angioplasty, 10% after atherectomy, and between 3.5% and 5% after stenting. Although antiproliferative drug-eluting stents help reduce restenosis by inhibiting neo-intimal growth, they can also lead to CAAs due to delayed reendothelialization, inflammatory changes in the medial wall, and hypersensitivity reactions [23,24,25,26].

Ahn et al. studied DES-associated CAAs in 3616 patients with a total of 4419 lesions who underwent follow-up angiography after DES implantation. They identified 34 CAAs (0.76% per lesion) in 29 patients (0.8% per patient) on average 414 ± 213 days after implantation. The current literature suggests that the incidence of CAAs associated with stent implantation ranges from 1.25% to 3.9%. However, this range may not accurately represent the true incidence due to limited data and variability in angiographic follow-up timing, CAA definitions, and inclusion criteria for post-PCI investigation.

Mechanisms proposed for stent-related CAA formation include coronary dissection and stent malapposition. The use of oversized, high-pressure balloons during PCI can lead to vessel dissection, and CAAs have been associated with bailout stenting following coronary dissection from PTCA [13,14]. While mechanical factors contribute to aneurysm formation after bare metal stent implantation, drug-eluting stents (DESs) introduce additional elements that can further compromise coronary vessels. DESs are coated with cytotoxic anti-restenosis drugs that inhibit smooth muscle and endothelial cell proliferation, thereby reducing restenosis. However, these antiproliferative properties also heighten the risk of CAAs by delaying neointimal healing and reendothelialization. Studies have shown that these drugs reduce neointimal formation, lead to persistent fibrin deposition, and cause macrophage infiltration, resulting in fewer smooth muscle cells in rabbit iliac and porcine coronary arteries at both 28 and 180 days post-implantation. Additionally, DESs can induce significant vascular inflammation, including increased eosinophilic infiltration, likely due to a hypersensitivity reaction to the drug-carrying polymer. These hypersensitivity reactions and the local cytotoxic effects of the drugs disrupt and weaken all three layers of the arterial wall, leading to expansion and increased susceptibility to aneurysmal disease [15,16,17]. Bioabsorbable vascular scaffolds (BVSs) used to gain attention in percutaneous coronary interventions (PCIs). The expansion of the vessel wall following BVS implantation can be influenced by several factors, including thorough lesion preparation, gradual degradation of the scaffold, eventual discontinuity of scaffold struts, and the resulting outward movement of the scaffold, which can lead to the formation of coronary artery aneurysms (CAAs). Further research is needed to accurately measure the incidence of CAAs and to better understand the underlying pathophysiological mechanisms associated with BVS implantation, as well as with other types of stents, to improve the management of these complications [18,19,20]—Table 2.

### 4.4. Molecular Mechanisms Linked to CAAs

Different studies have investigated the biological processes associated with differentially expressed proteins in coronary artery disease (CAD) and CAA. Notable differences were found between the two conditions. In CAD, there was a lower presence of proteins related to wounding response, lipoprotein modeling, platelet activation, and blood coagulation, suggesting stress responses linked to coronary artery injury. Conversely, in CAA patients, proteins associated with wounding and cholesterol components were significantly higher compared to controls, indicating more severe coronary artery damage.

The significant proteins identified were used to build a signaling network, revealing pathways enriched in infection, inflammation, and metabolism, aligning with previous hypotheses [27,28,29].

CFH and MBL2 are molecules involved in the complement system, which plays a crucial role in innate immunity. CFH regulates C3b production to prevent damage to host cells, while MBL2 aids in complement activation through the MBL pathway. Modified levels of CFH and MBL2 in KD with CAAs suggest complement system dysfunction.

KNG1 and SERPINC1 are part of the coagulation network. KNG1, interacting with HRG and coagulation factor XII, might affect blood coagulation. SERPINC1, a potent anticoagulant with anti-inflammatory properties, is downregulated in KD with CAA, which could contribute to increased thrombosis.

FN1, a multifunctional glycoprotein involved in cell adhesion, migration, wound healing, and blood coagulation, was reduced in both CAD and CAA. This decrease likely reflects endothelial dysfunction and stress responses in coronary artery lesions [30,31,32,33,34].

Histopathological analyses indicate that the destruction of coronary artery walls and diffuse vasculitis suggest that matrix metalloproteinases (MMPs) play a role in the formation of coronary artery aneurysms (CAAs). MMPs are enzymes that degrade connective tissue proteins, which weakens the vascular wall. Their activity is regulated by a prostaglandin E2–cAMP signaling pathway, which can be activated by inflammatory cytokines, leading to an imbalance in MMP levels. Smooth muscle cells produce tissue-specific MMP inhibitors, such as TIMP-1, TIMP-2, TIMP-3, and TIMP-4, to manage MMP activity. In aneurysmal vessels, there is often an increase in MMP-2, MMP-3, MMP-9, and MMP-12, alongside a reduction in TIMPs. This imbalance in proteolytic activity contributes to the degradation of the vessel wall matrix and the development of CAAs [35]

A study by Matsuyama et al. found that serum levels of MMP-3 and TIMP-1 are significantly higher in untreated Kawasaki disease (KD) patients compared to healthy children [36,37]. Furthermore, genetic factors such as variations in MMP-9 gene polymorphisms have been associated with aneurysm formation in KD [37]. Other genetic factors that may contribute to vessel wall weakening and the development of coronary artery ectasias include disruptions in the HLA-E and MMP-3 genes, as well as insertion/deletion polymorphisms in the angiotensin-converting enzyme (ACE DD genotype), SRC-1, and GRIN3A genes [38,39,40]—Table 3.

### 4.5. Disorders of the Connective Tissue and CAAs

Hereditary connective tissue disorders, such as Marfan syndrome and Ehlers–Danlos disease, can also induce coronary artery aneurysm (CAA) formation. Marfan syndrome is linked to mutations in the fibrillin 1 (FBN1) gene [20], which is essential for the microfibrils that support elastin fibers and maintain vessel wall integrity. The FBN1 gene interacts with latent transforming growth factor-beta (TGF-β)-binding proteins, keeping TGF-β in an inactive form. Mutations in FBN1 can lead to excessive TGF-β activity, potentially contributing to CAA formation through mechanisms such as cystic medial necrosis.

Additionally, mutations in the TGF-β receptor are associated with arterial aneurysms, and cystic medial necrosis is frequently observed in aortic aneurysms in individuals without Marfan syndrome, highlighting the role of TGF-β overactivity in CAA development.

Neurofibromatosis (NF) is a genetic disorder affecting neuroectodermal and mesodermal tissues, impacting the skin, nervous system, skeletal system, and vascular system. Although rare, there have been instances where NF patients have experienced myocardial infarctions due to thromboembolism [41].

### 4.6. Infections and CAAs

Various infections can serve as different etiologies for CAAs, including bacterial, mycobacterial, fungal, syphilitic, Lyme disease, septic emboli, and HIV infections. Each of these pathogens can contribute to the development of CAAs through different mechanisms. In some cases, direct invasion of pathogens into the vessel wall or deposition of immune complexes can drive the formation of mycotic CAAs. Specifically, mycotic aneurysms are often associated with infections caused by bacteria, fungi, or mycobacteria and can result from the direct infection of the vessel wall or through the effects of immune complex deposition. Syphilitic and Lyme disease infections can also lead to CAAs, as can septic emboli that lodge in the coronary arteries. In HIV-infected patients, CAAs may arise due to the complex interplay of immune suppression and opportunistic infections.

Recent studies suggest that drug-eluting stents (DESs) may also be implicated in the development of mycotic coronary aneurysms. While these devices are designed to reduce restenosis by releasing antiproliferative drugs, they can sometimes create a setting conducive to infection [42].

Mycotic aneurysms are often linked to infective endocarditis (IE) and can also occur in patients with sepsis, especially those who are immunocompromised. The most frequently identified pathogens responsible for mycotic aneurysms are *Staphylococcus aureus* and *Streptococcus viridans*. Other reported pathogens include *Salmonella* and *Pseudomonas*. Less common pathogens involved are coagulase-negative *Staphylococcus*, *Enterococcus*, *Candida* species, *Enterobacter*, *Mycobacterium bovis*, *Tuberculosis*, *Escherichia coli*, *Burkholderia*, and *Clostridium*. Recently, coronary stents have been increasingly associated with the development of mycotic coronary aneurysms, and the pathogens involved are similar to those found in IE [43,44,45].

Several pathophysiological mechanisms can explain the development of mycotic coronary aneurysms:

(a) Occlusion caused by embolization: Blockage of the arterial wall’s vasa vasorum, which can occur in an artery of normal caliber or with pre-existing aneurysm—this can cause an infarction of the vessel wall.
(b) Direct infiltration: Pathogens formed in a source of sepsis that is nearby and that can invade the arterial wall directly.
(c) Immune complex deposition: Damage caused by the deposition of immune complexes can injure different tunics of the vessel wall, leading to rapid enlargement of the wall and aneurysm formation.

In the context of infective endocarditis, mycotic aneurysms are thought to result from microemboli affecting the vasa vasorum or from local spread of infection from larger emboli originating at the primary infection site. For mycotic aneurysms related to intracoronary stent placement, multiple factors contribute, including contamination during stent delivery, transient bacteremia from skin flora via access-site hematomas, pseudoaneurysms, delayed bleeding, prolonged arterial sheath insertion, and multiple procedures through the same access site in a short period. Mycotic aneurysms secondary to systemic infection or sepsis are commonly associated with immunocompromised states, such as leukemia, AIDS, systemic lupus erythematosus (SLE), or renal transplant recipients. Additionally, patients requiring frequent vascular access, such as those on dialysis or receiving parenteral nutrition, are at increased risk of developing sepsis and, subsequently, mycotic aneurysms [46,47,48].

## 5. Discussion

Imaging technology is set to transform the way coronary artery aneurysms (CAAs) are diagnosed in the years to come. Improved magnetic resonance imaging (MRI) and higher-resolution coronary computed tomography angiography (CCTA) are two examples of novel imaging modalities that will provide more accurate and thorough evaluations of aneurysm morphology, size, and wall properties. These advancements may make it easier to identify minor aneurysms early on and offer more information about the likelihood of rupture or thrombosis. Furthermore, automatic detection and more precise classification of CAAs may be possible using artificial intelligence (AI) algorithms incorporated into imaging software, enhancing diagnostic efficiency and lowering human error. Through the analysis of massive datasets and the discovery of tiny patterns that are difficult to detect with existing approaches, AI may also help forecast the course of disease. New pharmacological treatments that target the molecular processes responsible for CAA development are being researched as a means of treatment. In the future, therapies might concentrate on reducing the inflammatory processes, endothelial dysfunction, and hereditary elements that lead to the formation of aneurysms. For example, patients with connective tissue disorders or aneurysms driven by atherosclerosis may benefit from anti-inflammatory medications, immunomodulatory medicines, or gene treatments, which have the ability to stop or reverse the growth of CAAs. Furthermore, improvements in antithrombotic and antiplatelet medications designed especially for aneurysmal illness may help patients with CAAs achieve a critical balance by lowering their risk of bleeding problems and thromboembolic consequences. For the purpose of treating or fixing CAAs, minimally invasive methods are expected to undergo significant advancements in the next ten years in both interventional and surgical approaches. Improved stent technology may lessen problems such as stent thrombosis or in-stent restenosis. Examples of this include bioresorbable or drug-eluting stents made especially for aneurysmal channels. Novel approaches in vascular grafting or vessel repair, such as the application of 3D-printed scaffolds or tissue-engineered grafts, may provide more long-lasting and specialized treatments for complicated aneurysms. As these therapies advance, more customized care plans that consider the unique risk factors and aneurysm characteristics of each patient are anticipated, which will eventually improve the long-term prognosis of CAA patients.

## 6. Conclusions

In recent years, our understanding of CAAs has advanced, but many aspects remain unclear. Nevertheless, some conclusions can be drawn:

- CAAs are rare but can lead to severe, potentially lethal outcomes.
- They are commonly related to CAD and are a concern due to percutaneous coronary interventions.
- Various mechanisms underlying CAA formation have been proposed, but precise processes remain elusive.
- Recent research has identified genetic polymorphisms that may increase the risk of developing CAAs, especially in conditions such as Kawasaki disease.
- Further research is needed to understand the risk factors and underlying mechanisms associated with CAAs, improving preventive and therapeutic strategies.

## Figures and Tables

**Table 1:** Comprehensive overview of the various causes and mechanisms of CAAs, along with associated risk factors.

Cause/Condition	Mechanism/Pathophysiology	Risk Factors
Atherosclerosis	Reduced tolerance to intraluminal pressure, leading to artery dilation; possible genetic predisposition (MMP3-5A allele) [8,9]	Chronic inflammation, high cholesterol, hypertension, smoking, diabetes
Congenital GCAAs	Developmental anomalies in coronary arteries leading to localized structural defects [9]	Genetic predisposition
Kawasaki Disease	Immune-mediated inflammation targeting arterial walls [10]	Genetic and environmental factors
Takayasu Arteritis	Systemic inflammatory process affecting large arteries [10]	Autoimmune conditions
Connective Tissue Diseases	(Marfan syndrome, Ehlers–Danlos syndrome, fibromuscular dysplasia, neurofibromatosis) [11]	Genetic disorders leading to weakened arterial walls
Other Vasculitis	(Lupus, polyarteritis nodosa, Behçet’s disease, rheumatoid arthritis, ankylosing spondylitis, scleroderma) [11,12]	Immune-mediated inflammation
Infections	(HIV, bacterial, mycobacterial, syphilis, Lyme disease, mycotic aneurysm, septic emboli) [12]	Infectious agents causing direct damage to arterial walls
Drug Use	(Cocaine, amphetamines, protease inhibitors) [10,11]	Direct toxic effects on arterial walls
Chest Trauma	Direct physical injury to coronary artery [10,11]	Blunt chest trauma
Tumors/Cardiac Lymphoma	Invasion and damage to coronary arteries [10,11]	Malignant growths
Percutaneous Coronary Intervention	Localized damage to arterial wall during procedure [13,14]	Chronic total occlusion, lesion length > 33 mm, lesion on the left anterior descending artery, implantation in infarct-related artery
Drug-coated Balloons	Localized arterial wall damage [15,16,17]	Interventional procedures
Bioresorbable Vascular Scaffolds	Remodeling processes post-implantation [18,19,20]	Interventional procedures
Rotablation	Mechanical damage to arterial wall [14,19,20]	Interventional procedures

**Table 2:** Main interventions contributing to CAAs.

Intervention	Contributing Factors	Aneurysm Occurrence Rate	Key Findings
Coronary Angioplasty	Persistent dissection, arterial barotrauma from high-pressure balloon inflation, and the use of oversized balloons [23]	3.9%	Aneurysm formation linked to high-pressure balloon inflation and dissection.
Atherectomy	Arterial barotrauma, atherectomy techniques [24,25]	10%	Higher rate of aneurysm formation compared to other interventions.
Stenting	Nonhealing dissection, stent malapposition, oversized stents [26]	3.5% to 5%	Incidence of CAAs in stenting ranges between 3.5% and 5%.
Drug-Eluting Stents (DESs)	Delayed reendothelialization, inflammatory changes, hypersensitivity reactions, reduced neointimal growth [13]	1.25% to 3.9%	DESs reduce restenosis but can increase CAA risk due to delayed healing and inflammatory responses. Reported CAA incidence is between 1.25% and 3.9%.
Bioabsorbable Vascular Scaffolds (BVSs)	Lesion preparation, scaffold degradation, strut discontinuity, outward scaffold displacement [18,19,20]	Not well defined; further research needed	Factors influencing CAA formation include thorough lesion preparation and scaffold degradation. Further research required to measure incidence and understand mechanisms.

**Table 3:** Molecular mechanisms of CAAs.

Aspect	Coronary Artery Aneurysm (CAA)
Protein Expression	Higher levels of proteins related to wounding response and cholesterol components compared to controls [27].
Signaling Network	Same pathways as CAD, with a focus on infection, inflammation, and metabolism [28,29].
CFH (Complement Factor H)	Plays a role in complement regulation. Reduced levels indicate dysfunction in the complement system [28,29].
MBL2 (Mannose-Binding Lectin 2)	Participates in complement activation. Decreased levels in KD with CAAs suggest dysfunction in the complement system [29].
KNG1 (Kininogen 1)	Downregulated in KD with CAA, possibly contributing to increased thrombosis [30].
SERPINC1 (Antithrombin III)	Downregulated in KD with CAA, which might increase thrombosis risk [30].
FN1 (Fibronectin 1)	Reduced in both CAD and CAA, indicating endothelial dysfunction and stress responses in coronary artery lesions [31].
Histopathological Findings	Destruction of coronary artery walls and diffuse vasculitis suggest involvement of matrix metalloproteinases (MMPs) [31].
MMPs (Matrix Metalloproteinases)	Increased levels of MMP-2, MMP-3, MMP-9, and MMP-12, along with decreased TIMPs. This imbalance leads to vessel wall degradation and CAA formation [32].
Tissue-Specific Inhibitors (TIMPs)	Decreased TIMP-1, TIMP-2, TIMP-3, and TIMP-4 levels in aneurysmal vessels, contributing to proteolytic imbalance [33].
Genetic Factors	MMP-9 gene polymorphisms linked to aneurysm formation in KD. Other associated factors include HLA-E, MMP-3 gene disruptions, ACE DD genotype, SRC-1, and GRIN3A genes [33].
